# The potential effects of DPP‐4 inhibitors on cardiovascular system in COVID‐19 patients

**DOI:** 10.1111/jcmm.15674

**Published:** 2020-07-26

**Authors:** Hengzhi Du, Dao Wen Wang, Chen Chen

**Affiliations:** ^1^ Division of Cardiology Department of Internal Medicine Tongji Hospital Tongji Medical College Huazhong University of Science and Technology Wuhan China; ^2^ Hubei Key Laboratory of Genetics and Molecular Mechanisms of Cardiological Disorders Wuhan China

**Keywords:** cardiovascular system, COVID‐19, DPP‐4 inhibitors

## Abstract

With the outbreak of a new coronavirus, severe acute respiratory syndrome coronavirus 2 (SARS‐CoV‐2), the public healthcare systems are facing great challenges. Coronavirus disease 2019 (COVID‐19) could develop into severe pneumonia, acute respiratory distress syndrome and multi‐organ failure. Remarkably, in addition to the respiratory symptoms, some COVID‐19 patients also suffer from cardiovascular injuries. Dipeptidyl peptidase‐4 (DPP‐4) is a ubiquitous glycoprotein which could act both as a cell membrane‐bound protein and a soluble enzymatic protein after cleavage and release into the circulation. Despite angiotensin‐converting enzyme 2 (ACE2), the recently recognized receptor of SARS‐CoV and SARS‐CoV‐2, which facilitated their entries into the host, DPP‐4 has been identified as the receptor of middle east respiratory syndrome coronavirus (MERS‐CoV). In the current review, we discussed the potential roles of DPP‐4 in COVID‐19 and the possible effects of DPP‐4 inhibitors on cardiovascular system in patients with COVID‐19.

## 1 INTRODUCTION

Since early December 2019, the outbreak of the coronavirus disease 2019 (COVID‐19) occurred. With the rapid spread in the world, the number of infected cases has grown exponentially. Recently, the development in the detection technologies of severe acute respiratory syndrome coronavirus 2 (SARS‐CoV‐2) has given rise to a better understanding of the clinical characteristics and molecular epidemiology of COVID‐19. It is noteworthy that according to the present clinical data, cardiac injury is one of the most common complications in patients with COVID‐19, which is also associated with poor prognosis.^1^

Dipeptidyl peptidase‐4 (DPP‐4) is a widely expressed glycoprotein that could not only act as a cell membrane‐bound receptor but also as a soluble enzymatic protein. The enzymatic functions of DPP‐4 were well‐recognized. DPP‐4 has a variety of substrates, including incretin hormones, cytokines, chemokines, neuropeptides and growth factors. DPP‐4 is widely expressed in the blood vessels, myocardium and myeloid cells. It has been reported that the polymorphisms of DPP‐4 were associated with the risk of myocardial infarction in patients with atherosclerosis.^2^

Previous studies showed that membrane‐associated human DPP‐4 was a functional receptor of middle east respiratory syndrome coronavirus (MERS‐CoV), which interacted with MERS‐CoV through the spike glycoprotein S1b domain to promote viral entry.^3^ Moreover, by blocking spike protein S1 binding to DPP‐4, recombinant human adenosine deaminase (ADA) could inhibit MERS‐CoV infection of cells transfected with human DPP‐4.^4^ Considering the similar outer membrane spike glycoproteins among the coronavirus, it is possible that DPP‐4 might also be a functional receptor of SARS‐CoV‐2. Like angiotensin‐converting enzyme 2 (ACE2), DPP‐4 played important roles in cardiovascular physiological processes and metabolism homeostasis. Here, we review the potential roles of DPP‐4 in COVID‐19, especially in the cardiovascular injury of COVID‐19 patients.

## 2 HUMAN DPP‐4 MIGHT BE A SARS‐CoV‐2 RECEPTOR

DPP‐4 could present as a membrane binding protein or a cleaved soluble enzyme protein. It has been reported that membrane‐associated human DPP‐4, as a functional MERS‐CoV receptor, interacted with MERS‐CoV through the spike glycoprotein S1b domain to facilitate the entry of MERS‐CoV.^3^ Moreover, blocking spike protein S1 or the receptor‐binding domain (RBD) of the MERS‐CoV Spike protein could directly against MERS‐CoV binding to human DPP‐4, thereby prevent MERS‐CoV infection.^5^ Considering the similarity among MERS‐CoV and the other coronavirus, it has been speculated that membrane‐associated human DPP‐4 might also be a functional SARS‐CoV‐2 receptor.^6^ Moreover, Naveen et al detected that the S1 domain of SARS‐CoV‐2 spike glycoprotein, the key immunoregulatory factor for hijacking and virulence, potentially interacted with the human DPP‐4 by overall homo‐trimer model structure.^7^ However, whether DPP‐4 is indeed a direct receptor of SARS‐CoV‐2 remains to be further verified.

## 3 THE ROLE OF DPP‐4 IN CARDIOVASCULAR INJURY

One of the major functions of DPP‐4 is degrading incretin hormones, including incretin hormones (glucagon‐like peptide‐1 [GLP‐1] and gastric inhibitory polypeptide [GIP]), cytokines, chemokines, neuropeptides and growth factors.^8^ It is well‐known that GLP‐1 and GIP promote insulin secretion from the pancreatic β cells and suppress glucagon secretion from other cells. Therefore, the inhibitors of DPP‐4 are widely used to treat diabetes. Moreover, further studies find that DPP‐4 is also involved in the cardiovascular complications of diabetes.

In atherosclerosis, the key pathogenesis is chronic inflammation characterized by accumulation of plaques within the arteries.^9^ It was discovered that DPP‐4 enhanced monocytes migration to atherosclerotic plaque and down‐regulated the expression of adiponectin, which promoted inflammation and the formation of atherosclerotic plaques.^10^ Moreover, DPP‐4 could down‐regulate stromal‐derived factor 1 (SDF‐1), a chemoattractant for multiple cell types,^11^ while inhibition of SDF‐1‐mediated chemical protection and proliferation of hematopoietic stem cells and progenitor cells would inhibit neovascularization and the recovery of tissue damage.^12^ In vein endothelial cells, by inhibiting the GLP‐1R signalling pathway, DPP‐4 could also promote the development of atherosclerosis.^13^

In addition to atherosclerosis, DPP‐4 also plays a negative role in the process of heart failure. DPP‐4 could degrade brain natriuretic peptide (BNP), which is secreted from the cardiac ventricles in response to stretch,^14^ into a less potent metabolite BNP (3‐32), resulting in a loss of the BNP‐mediated protective effects on the heart. Moreover, DPP‐4 could also inhibit the activation of GLP‐1R, which localized in the cardiac atria, reduce the secretion of atrial natriuretic peptide and increase the blood pressure.^15^ Thus, DPP‐4 inhibitor therapy may have additional favourable influences on cardiovascular system.

## 4 THE POTENTIAL EFFECTS OF DPP‐4 ON CARDIOVASCULAR SYSTEM IN COVID‐19

As mentioned above, DPP‐4 might be the functional SARS‐CoV‐2 receptor which facilitates the entry of SARS‐CoV‐2 into the host cells, including cardiomyocytes. Although the presence of SARS‐CoV‐2 in the heart was confirmed by biopsy, as well as myocarditis was observed in certain COVID‐19 patients, the underlying mechanism of SARS‐CoV‐2‐induced cardiac injury still needs further investigation. SARS‐CoV‐2 might damage the cardiomyocytes directly, and the cardiomyocytes might be injured indirectly via the systemic cytokine storm or the interactions between organs.

Evidence from severely ill patients with COVID‐19 suggested that the release of cytokines and chemokines was delayed in respiratory epithelial cells, dendritic cells (DCs) and macrophages at the early stage of SARS‐CoV‐2 infection.^16^ Later, it was found that high levels of pro‐inflammatory cytokines (IL‐6, IL‐10 and TNF‐α), lymphopenia (reduced CD4^+^ and CD8^+^ T cells) and low levels of antiviral factors (interferon, IFNs) were positively associated with the severity of COVID‐19.^17^ Similar findings were also observed in SARS‐CoV and MERS‐CoV infected human airway epithelial cells, THP‐1 cells, human peripheral blood monocyte‐derived macrophages and DCs.^18^ Although inflammation initially only damages limited organs, such as the lungs, an over‐activated inflammatory response will spread all over the body rapidly, including the heart. This maybe one of the reasons why the plasma levels of cardiac injury biomarkers usually positively correlated with the plasma levels of inflammatory markers.

**Figure 1:**
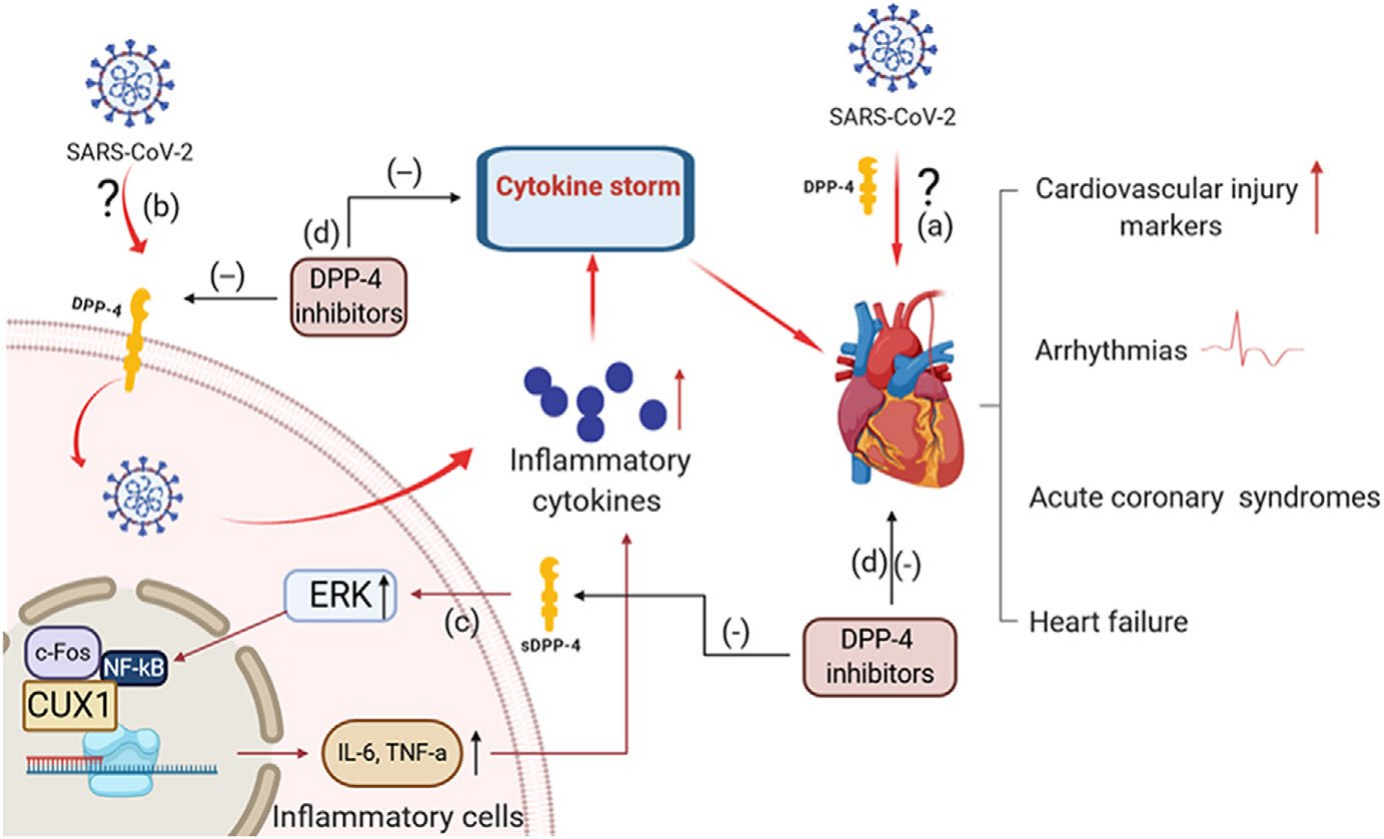
The potential roles of DPP‐4 in COVID‐19 related cardiovascular injury. A, DPP‐4 might promote the production of inflammatory storm mediated by SARS‐CoV‐2 and promote damage to the cardiovascular system. B, DPP‐4 might be the functional SARS‐CoV‐2 receptor which facilitates entry into host cells, assisting the direct myocardial injury of SARS‐CoV‐2. C, DPP‐4 up‐regulated ERK in the cytosol and c‐Fos, NF‐kB p65, NF‐kB p50 and CUX1 in the nucleus enhance the expressions of TNF‐α and IL‐6 in a DPP‐4 enzyme activity‐dependent manner. D, DPP‐4 inhibitors might alleviate the COVID‐19 related cardiovascular injury. CUX1, CUT‐like homeobox 1; ERK, extracellular signal‐regulated kinase; NF‐kb, nuclear factor‐kappa B

During the infection of SARS‐CoV‐2, DPP‐4 might play important roles. First, in MERS‐CoV related studies, Ahmed et al found that spike glycoprotein MERS‐CoV suppressed macrophage responses via DPP‐4‐mediated induction of interleukin‐1 receptor‐associated kinase (IRAK)‐M and peroxisome proliferators‐activated receptor γ (PPARγ) at the early stage of infection.^19^ They also demonstrated that indeed the immunosuppressive effects of the S glycoprotein could be ameliorated by sitagliptin, a DPP‐4 inhibitor, via down‐regulating IRAK‐M and PPARγ. Meanwhile, Tetsurou et al found that DPP‐4 could enhance the transcription of IL‐6 and TNF‐α in THP‐1 cells and monocytes.^20^ They also found that stimulation with a combination of DPP‐4 and LPS would up‐regulate ERK in the cytosol and c‐Fos, NF‐kB p65, NF‐kB p50 and CUX1 in the nucleus, which enhanced the transcription of TNF‐α and IL‐6 in a DPP‐4 enzyme activity‐dependent manner (Figure 1). Moreover, Hiromura et al demonstrated that DPP‐4 exerted a pro‐inflammatory effect in mouse and human macrophages, and caveolin‐1, a binding protein of DPP‐4, was essential for the anti‐inflammatory effects of DPP‐4 inhibitors.^21^

Proper inflammation is an essential part of an effective immune response which eliminates the pathogens and ultimately leads to tissue repair and restoration of homeostasis. However, SARS‐CoV‐2 might induce excessive and prolonged cytokine responses in severely ill cases with ARDS or MODS, even death. Although the direct involvement of DPP‐4 in SARS‐CoV‐2 infection needs to be clarified, there is evidence suggesting that DPP‐4 inhibitors might be of potential benefits in patients with COVID‐19.

## 5 THE FUNCTION OF DPP‐4 INHIBITORS IN COVID‐19

Early control of cytokine storms through immunomodulators, cytokine antagonists or immunoadsorption to reduce the infiltration of inflammatory cells is the key to improve the prognosis and reduce the mortality of COVID‐19 patients.

DPP‐4 inhibitors, such as sitagliptin, alogliptin, vildagliptin, saxagliptin and linagliptin, which selectively inhibit the catalytic activity of cell‐associated and circulating soluble DPP‐4, are widely used drugs against diabetes. DPP‐4 inhibitors may be of potential use for severe COVID‐19 by suppressing T cell proliferation and the production of pro‐inflammatory cytokines.^22^ For example, Takeshi et al^23^ showed that sitagliptin alleviated lung injury by inhibiting pro‐inflammatory cytokines IL‐1β, TNF‐α and IL‐6 in an experimental model of ARDS, which was also the main cause of SARS‐CoV‐2‐induced death. Ta et al^24^ detected that alogliptin reduced Toll‐like receptor 4 (TLR4)‐mediated up‐regulation of IL‐6 and IL‐1β in diabetic ApoE^−/−^ mice. Moreover, sitagliptin interacted with one of the predicted binding sites (V341) of SARS‐CoV‐2, which might modify the SARS‐CoV‐2‐DPP‐4 interaction.^7^ In addition, previous studies revealed that DPP‐4 inhibitors could improve cardiovascular function directly.

It was worth mentioning that some reports published recently showed that the role of DPP‐4 inhibitors in type 2 diabetes (T2D) patients with COVID‐19 was controversial. Eleftheriou et al^25^ suggested that DPP‐4 inhibitors might be beneficial to COVID‐19 infections with diabetes. In addition, Scheen et al suggested that no negative sign was identified regarding the use of DPP‐4 inhibitors and the outcome of COVID‐19 patients.^26^ Moreover, by retrieving information on exposure to DPP‐4 inhibitors among patients with diabetes hospitalized for COVID‐19 at an outbreak hospital in Italy, Fadini et al found no evidence that DPP‐4 inhibitors may be more useful for infected patients with diabetes.^27^ Bouhanick et al^28^ suggested that DPP4 inhibitors should be used with caution or discontinued upon admission to hospital of unstable patients and critically ill patients. Although the reported effects of DPP‐4 on COVID‐19 patients with diabetes are controversial, these studies mainly focus on the mortality and did not assess the potential effects of DPP‐4 inhibitors on cardiovascular injury in COVID‐19 patients.

Taken together, DPP‐4 might be a functional SARS‐CoV‐2 receptor which facilitates viral entry into host cells, assisting the direct myocardial injury or inflammatory storm production mediated by SARS‐CoV‐2. Moreover, DPP‐4 inhibitors might alleviate the COVID‐19 related cardiovascular injury including arrhythmia, acute coronary syndrome and heart failure (Figure 1).

## 6 CONCLUSION

In this review, we put forward a hypothesis that DPP‐4 might participate in the process of SARS‐CoV‐2 infection. This may promote COVID‐19 progression towards an hyperinflammatory state, which could damage the cardiovascular system. Meanwhile, DPP‐4 inhibitors could inhibit the over‐activated inflammatory caused by SARS‐CoV‐2 and thus improve cardiovascular function. Moreover, more clinical and laboratory evidence about the effects of DPP‐4 inhibitors on COVID is urgently needed.

## CONFLICTS OF INTEREST

None.

## AUTHOR CONTRIBUTION

**Hengzhi Du:** Conceptualization (equal); Investigation (lead); Writing‐original draft (lead). **Dao Wen Wang:** Funding acquisition (lead); Supervision (equal). **Chen Chen:** Conceptualization (lead); Supervision (lead); Writing‐review & editing (lead).
